# Collagen and hyaluronan at wound sites influence early polymicrobial biofilm adhesive events

**DOI:** 10.1186/1471-2180-14-191

**Published:** 2014-07-16

**Authors:** Eric Birkenhauer, Suresh Neethirajan, J Scott Weese

**Affiliations:** 1 BioNano Laboratory, School of Engineering, University of Guelph, Guelph, Ontario N1G 2 W1, Canada; 2 Department of Pathobiology, Ontario Veterinary College, University of Guelph, Ontario N1G 2 W1, Canada

**Keywords:** *Pseudomonas aeruginosa*, MRSA, Polymicrobial biofilms, Collagen, Hyaluronan, Microtiter plate assay, Chronic wound infections, Wound biofilms, Atomic force microscopy

## Abstract

**Background:**

Wounds can easily become chronically infected, leading to secondary health complications, which occur more frequently in individuals with diabetes, compromised immune systems, and those that have suffered severe burns. When wounds become chronically infected, biofilm producing microbes are often isolated from these sites. The presence of a biofilm at a wound site has significant negative impact on the treatment outcomes, as biofilms are characteristically recalcitrant to removal, in part due to the formation of a protective matrix that shield residents organisms from inimical forces. *Pseudomonas aeruginosa* and methicillin-resistant *Staphylococcus aureus* (MRSA) are two of the organisms most prevalently isolated from wound sites, and are of particular concern due to their elevated levels of antibiotic resistance, rapid growth, and exotoxin production. In order to understand the biofilm forming abilities of these microbes in a simulated wound environment we used a microtiter plate assay to assess the ability of these two organisms to bind to proteins that are typically found at wound sites: collagen and hyaluronan.

**Results:**

Collagen and hyaluronan were used to coat the wells of 96-well plates in collagen:hyaluronan ratios of 0:1, 3:1, 1:1, 1:3, and 1:0 . *P. aeruginosa* and MRSA were inoculated as mono- and co-cultures (1:1 and a 3:1 MRSA: *P. aeruginosa*). We determined that coating the wells with collagen and/or hyaluronan significantly increased the biofilm biomass of attached cells compared to an uncoated control, although no one coating formulation showed a significant increase compared to any other combination. We also noted that the fold-change increase for MRSA upon coating was greater than for *P. aeruginosa*.

**Conclusions:**

Our study suggests that the presence of collagen and/or hyaluronan at wound sites may be an important factor that influences the attachment and subsequent biofilm formation of notorious biofilm-formers, such as MRSA and *P. aeruginosa*. Understanding the kinetics of binding may aid in our comprehension of recalcitrant wound infection development, better enabling our ability to design therapies that would prevent or mitigate the negative outcomes associated with such infections.

## Background

The treatment and management of chronic wounds represents a significant burden to both healthcare systems and patients [1-3]. The treatment of chronic wounds is costly; treatment of leg ulcers in North America can cost up to US$ 45, 000 for patients [3]. This does not account for deleterious effects on the quality of the patient’s life [3]. Most chronic wounds arise from amputation, burns, or venous ulcers and can become exacerbated in the case of immunocompromised, obese, or diabetes mellitus patients [4-6]. Underlying layers of cutaneous tissue can become exposed to opportunistic and pathogenic microbes at wound sites. The release of the cytoplasmic contents from damaged cells makes this a nutrient-rich environment that is highly susceptible to infection from the patient’s endogenous microbiota, the environment or from healthcare providers [7].

Within the last 20 years, it has become widely accepted that many organisms exist as a biofilm community, which diverges from earlier microbial theorem that postulates microorganisms operate as individual cells [8]. Today, we recognize that the vast majority of microbes are found in dynamic complex communities, which include polymicrobial biofilms [9-11]. Common constituents of polymicrobial wound biofilms include methicillin-resistant *Staphylococcus aureus* (MRSA) and *Pseudomonas aeruginosa* with many studies examining the dynamics of these microbes [12,13]. MRSA and *P. aeruginosa* readily colonize wound sites, forming biofilms that are resistant to antibiotics, phagocytes, and other immune system components, such as antibodies and complement proteins [4-7,14,15]. Recently, *S. aureus* and *P. aeruginosa* have been implicated in producing and secreting human surfactant proteins which may explain their aggressive colonization and biofilm production in the lungs of cystic fibrosis patients [16].

*P. aeruginosa* is a prolific biofilm former, producing biofilms within as little as 10 hours *in vitro*[17]. Studies show that the major mechanism of attachment involves type IV pili; cells initially attach reversibly via pili and eventually become irreversibly attached, leading to the loss of pili [8]. *P. aeruginosa* is a common inhabitant of soil environments and can be found on the skin [13]. Although it is commonly not harmful to healthy individuals, it is a voracious opportunistic pathogen with a high-affinity for water and will frequently occupy mucosal surfaces [13,18-20]. *P. aeruginosa* has been implicated in less severe cases of dermatitis, but infection may become serious in cutaneous infections of immunocompromised patients [13]. In microbial biofilm communities, *P. aeruginosa* increases biofilm virulence by cooperatively helping the growth of other microorganisms [13,21]. *P. aeruginosa* is a siderophore-producing bacterium, which allows it to scavenge iron, a growth limiting nutrient, from erythrocytes present at wound sites [21]. In many cases, non-siderophore producing microorganism, such as *S. aureus*, are able to scavenge *P. aeruginosa*’s siderophores, leading to higher levels of microbial growth [21].

*Staphylococcus aureus* is a transient colonizer of the nasal passages and is carried asymptomatically in the nasopharynx of 35% to 60% of the human population [13]. It too, like *P. aeruginosa,* is an opportunistic pathogen and the causative agent of many self-limiting skin infections [13]. However, if infection from *S. aureus* persists, more serious complications may occur such as cellulitis, bacteremia, pneumonia, and toxic shock. The most common sites of *S. aureus* infection include the skin and soft tissues [13]. To that effect, 75% of soft tissue infections are caused by the methicillin-resistant *Staphylococcus aureus* (MRSA), *S. aureus* that is resistant to all β–lactam antibiotics by virtue of acquisition of *mecA* and which often acquires resistance to other antimicrobial classes [13,22]. MRSA is non-motile and does not produce surface appendages, such as pili (as observed with *P. aeruginosa*). Instead MRSA uses surface proteins such as ClfB and Eap and relies on surface charges in order to mediate adherence to squamous epithelial cells [23-25].

In order to better understand how *P. aeruginosa* and MRSA form biofilms at wound sites, it is critical to understand the overall dynamics of the wound environment. Collagen and hyaluronan are two common proteins found in the skin, and both excellent candidates for the generation of skin substitutes [26,27]. Both are found in the dermal region of the skin, which is mostly acellular, consisting of collagen, elastin, and hyaluronan (among other glycosaminoglycans) [28]. This is the opposite of the epidermis, which is composed of up to 95% keratinocytes [28]. Collagen, elastin, and hyaluronan compose the dermal extracellular matrix in which fibroblasts and macrophages reside [28]. Collagen is the main component of connective tissue and functions as a support for tissue growth and acts as a chemo-attractant and scaffold for keratinocyte and fibroblast cells [29,30]. Hyaluronan is a common component of all extracellular matrices and is involved in regulation of cell function, migration, proliferation, and differentiation [31].

The collagen and hyaluronic acid ratio vary between individual’s age and sex. Collagen accounts for 70% of dermis [32], while hyaluronic acid forms a smaller part of the dermis. The amount of hyaluronic acid is about 0.1 to 0.2 microgram/milligram dry weight of the dermis [32]. Collagen is cationic but hyaluronan is anionic, and hence the two macromolecules may form polyionic complexes in aqueous solution [33]. Higher hyaluronic acid concentration in the cross-linked collagen-hyaluronan matrix may lead to higher stiffness and higher bulk modulus in the skin.

Due to their likely importance in chronic wound infections, we examined the interactions among collagen, hyaluronan, MRSA, and *P. aeruginosa*. As anticipated, we found that the presence of collagen and hyaluronan influences the early adhesive events that are critical for the establishment of potentially life threatening MRSA and P. *aeruginosa* infections. Ultimately, we found that although differences in the formulations of these components did not affect biofilm formation, the presence of any formulation (each alone or together in different ratios) significantly increased the biofilm biomasses of both MRSA and *P. aeruginosa*. Our results support the notion that collagen and hyaluronan may be important ligands for microbial attachment. Understanding the dynamics of binding may lead to improved therapies that target adhesion.

## Methods

### Bacterial strains

Bacterial strains used in this study consisted of MRSA and *Pseudomonas aeruginosa*. Two strains of each microbe were used. *P. aeruginosa* BK-68 and *P. aeruginosa* BK-76 were obtained from canine ear skin infections. MRSA M05-35 (USA 100) and MRSA M05-86 (USA 300) were obtained from human skin infections.

### Culture conditions

MRSA and *P. aeruginosa* strains were streaked onto 5% sheep blood agar (SBA) plates from −80°C frozen stocks, and were grown inverted at 37°C for 24 hours. For microtiter plate and AFM experiments, cultures were grown in 5 mL tryptic soy broth (TSB) at 37°C for 24 hours.

## Microtiter plate assay

### Collagen and hyaluronan coating

Standard 96-well polystyrene microtiter plates were coated with rat-tail collagen type I (BD Biosciences) and hyaluronan (0.05 mg/ml, Sigma) both dissolved in 0.9% NaCl solutions at concentrations of 50 μg/mL, similar to the protocol carried out by Werthen et al. [34]. Wells consisted of 1:0, 3:1, 1:1, 1:3, and 0:1 collagen:hyaluronan coatings in replicates of four along with a replicates of uncoated wells. To each well, a total of 300 μL of solution was added. Plates were then covered and allowed to incubate at 4°C for 24 hours. Coating solutions were gently removed after this time and the wells were rinsed twice with a 0.9% NaCl solution.

### Inoculation and incubation

Single colonies from SBA plates were cultured in 5 mL of TSB and were allowed to grow for 24 hours at 37°C in a reciprocal shaker (200 rpm). Cultures were standardized to ~1.5 × 10^8^ CFU/ml in TSB (supplemented with 0.1% NaCl) using a 0.5 McFarland standard. For mono-cultures, 200 μL was inoculated in replicates of four to the coated and uncoated wells. For 1:1 co-cultures, 100 μL of MRSA and 100 μl of PA were inoculated in replicates of four to the coated and uncoated wells. For the 3:1 MRSA:PA co-culture, 150 μL of MRSA and 50 μL of PA were inoculated in replicates of four to the coated and uncoated wells. Controls were prepared and incubated alongside the microtiter plate assay and underwent the same subsequent treatments (heat-fixing, crystal violet staining, etc.). No contamination was apparent in the negative controls (no opacity/turbidity). Average OD values obtained from the controls were subtracted from the corresponding experimental values obtained. Microtiter plates were incubated for 24 hours at 37°C under static conditions in order to allow for biofilm formation [34,35].

### Quantification of biofilms

The quantification of biofilms was done using a modified protocol of Singh et al. [36]. After 24 hour growth in microtiter plates, microbial solutions were discarded. Wells were washed three times with 200 μL PBS (pH = 7.2) in order to remove unattached cells. Biofilms were then heat-fixed at 60°C for 60 minutes and were subsequently stained with 0.1% crystal violet for 15 minutes at room temperature. Crystal violet was aspirated with a pipette, and the plates were rinsed by submersion in a container of tap water. Plates were then allowed to dry for 60 minutes at 35°C. After this the stained biofilms were re-solubilized in 200 μL of 95% ethanol and OD was taken at 562 nm.

### Selective dilution plating

In order to enumerate the number of viable microbial cells in mono- and co-cultures, selective dilution plating experiments were performed. Mono-cultures were grown in TSB (supplemented with 0.1% NaCl) for 24 hours at 37°C in a reciprocal shaker (200 rpm). Mono-cultures were then standardized to a 0.5 McFarland standard. 1 mL of each standardized mono-culture was then inoculated into separate test tubes containing 6 mL of fresh TSB broth. For the 1:1 co-culture, 1 mL of each mono-culture was inoculated into the same test tube containing 6 mL of fresh TSB. For the 3:1 MRSA:PA co-culture, 1.5 mL and 0.5 mL of MRSA and *P. aeruginosa* strains were inoculated into 6 mL of fresh TSB. These three cultures were then grown for 24 hours under the same conditions as previously mentioned. After 24 hours, 1 mL from each culture (PA-76, MRSA-35, and PA-76 + MRSA-35) was inoculated into separate 9 mL PBS (pH = 7.4) test tubes to create 10^−1^ dilutions. From this, 1 mL was transferred to 9 mL of PBS to create the next dilution. This process was repeated until 10^−7^ dilutions were generated. 0.1 mL from each dilution was then plated and grown for 24 hours at 37°C. Mono-cultures were plated on 5% SBA. Co-cultures were plated on Pseudomonas CFC Agar (Oxoid) and Staphylococcus Medium 110 (Oxoid) in order to select for *P. aerugionsa* and MRSA respectively. Plates containing 25–250 colonies were used in determining bacterial CFU/mL.

### Atomic force microscopy

Single colonies of MRSA M05-35 and PA BK-76 were inoculated into separate test tubes containing 5 mL TSB each. A co-culture was generated by inoculating single colonies of these strains into the same test tube containing 5 mL TSB. These were grown for 24 hours at 37°C. After this time 1 mL from each culture was centrifuged at 1200 rpm for 3 minutes and washed twice with deionized water. 200 μL of the washed cell solutions was pipetted onto mica sheets pre-coated with gelatin (0.005 g/mL). A gelatin pre-coating was done in order to improve cell to surface attachment [37]. Cultures were allowed to sit on the mica sheets for 30 minutes before being rinsed off with 1 mL of deionized water. After rinsing, the mica sheets were covered and allowed to dry overnight. Imaging was done using the intermittent-contact mode (AC mode) in air.

### Negative controls

Negative controls for the microtiter plate assay consisted of inoculating TSB (replicates of four) into the coated (collagen:hyaluronan in varying ratios as describe above) and uncoated wells. For the selective dilution plating, 0.1 mL of the PA-76 and MRSA-35 (100 dilutions) were inoculated onto Staphylococcus Medium 110 and Pseudomonas CFC Agar, respectively. No growth was observed after 24 hours at 37°C.

### Statistical methods

Experiments were conducted in triplicate and repeated twice. A student’s t-test was performed to compare groups with a P < 0.05 being considered significant. Statistical analysis was performed on commercially available software (R Open Source Statistical Programming).

## Results and discussion

### Coating with collagen and hyaluronan increases *P. aeruginosa* and MRSA attachment

**Figure 1:**
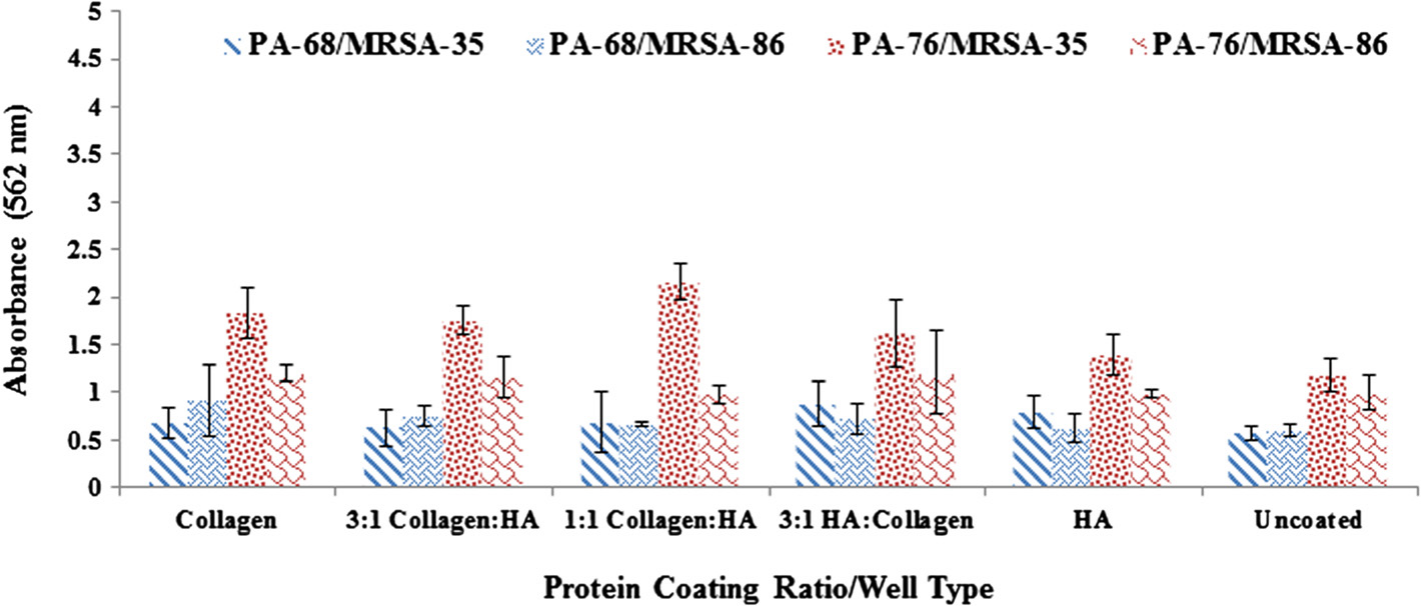
**Collagen and Hyaluronan enhance Biofilm Production in *P. aeruginosa *and MRSA Mono-cultures.** Biofilm production as observed at OD = 562 nm after crystal violet microtiter plate assay among *P. aeruginosa* (PA-68, PA-76) and MRSA (MRSA-35, MRSA-86). The various protein ratios/well coatings are shown in the x-axis with HA representing hyaluronan. Error bars shown represent the 1 standard deviation values for each sample.

When we examined *P. aeruginosa* BK-68 (PA-68) biofilm growth on coated vs. uncoated surfaces, we found that coated wells harbored more biomass after a 24 h incubation; however, not to a significant extent in most cases with the exceptions being the 3:1 hyaluronan:collagen and the collagen coatings (P = 0.02 and P = 0.02 respectively) (Figure 1). Among the coated wells, the 3:1 collagen: hyaluronan well led to the highest biofilm growth with an absorbance of 1.73. The lowest absorbance among the coated wells occurred in the 1:1 collagen: HA coated well, with an absorbance reading of 1.39.

Similar to strain PA-68, *P. aeruginosa* BK-76 (PA-76) biofilm production was again enhanced in coated wells; however, not to a significant extent in most cases. Exceptions here this time were seen with the collagen and hyaluronan coated wells (P = 0.03 and P = 0.04 respectively). The greatest production occurring in the collagen coated well (Abs = 3.71). The lowest biofilm production in the coated wells was observed in the 1:1 collagen: HA well (Abs = 2.66), similar to PA-68. Between well coatings, no significant differences in biofilm biomass production were observed in both *P. aeruginosa* strains. This suggests that none of the coatings combinations in particular promoted greater or lesser biofilm biomass.

Coatings also increased biofilm formation in MRSA M05-35 (MRSA-35) and MRSA M05-86 (MRSA-86) (Figure 1) after 24 h of growth; however, in this case all coating showed a significant increase in biofilm formation compared to the uncoated wells (P < 0.05). For both MRSA strains, the collagen wells showed the greatest significant difference compared to the uncoated wells (P < 0.001 in both cases). For MRSA-35 all collagen:hyaluronan wells showed significantly less biofilm biomass than the hyaluronan coated well (P < 0.05). This was not observed when comparing the collagen and hyaluronan coated wells with the collagen well showing greater biofilm biomass production, but not to a significant extent. Among well coatings for MRSA-86, a significant decrease in biofilm biomass was observed in 1:1 collagen: hyaluronan and 3:1 hyaluronan:collagen wells compared to the collagen well (P = 0.002 and P = 0.005 respectively).

Ultimately, we determined that collagen coating by itself appears to be the most effective substrate for biofilm attachment. We found it interesting that HA alone or the addition of HA to collagen did little to alter the biofilm attachment phenotype, considering that it is frequently present at the wound site [38]. This data suggests that these important wound infection pathogens (particularly MRSA) may have a preference for binding to skin and wound proteins [34]. At this time, we do not know if that is preferential binding is dictated by specific ligands or whether the interaction is governed by the intrinsic physical properties of collagen.

The significant increase in biofilm production from both MRSA strains between coated and uncoated wells is believed to be due to MRSA’s less efficient ability, compared to *P. aeruginosa*, to attach and adhere to surfaces, a vital component in the formation of biofilms [12,39-41]. The enhancement in biofilm formation upon coating of the wells was less dramatic for *P. aeruginosa*. We believe this may be because *P. aeruginosa* is efficient at adhering to nearly any surface, including polystyrene [40,42,43]. *P. aeruginosa* is known to have more effective methods of attachment and adherence to surfaces, most notably through type IV pili (TFP), whereas *S. aureus* relies mainly on surface protein-protein interactions for attachment [23,24,41,44-46]. Specifically Eap, ClfB, and teichoic acid, all of which are *S. aureus* membrane proteins, have been implicated as key proteins for initial attachment [23,24,41]. Other adhesive factors associated with Staphylococcal microbial attachment include eDNA, accumulation-associated proteins (Aaps), phenol-soluble modulins (PSMs), polysaccharide intracellular adhesins (PIAs), and microbial surface components recognizing adhesive matrix molecules (MSCRAMMs) [47]. The net cell surface charge of *S. aureus*, as well as surface porosity itself has been shown to significantly affect initial cell attachment [40,41]. It was shown that among MRSA mono-cultures, any coating combination helped improve the attachment and adherence. This is significant as these proteins are commonly found in the natural wound environment and have previously been shown to promote attachment and adherence of many microorganisms [34,38].

PA-68 and PA-76 are relatively newly isolated strains and have not yet been fully characterized through genomic and proteomic analysis. The difference in the binding activity of the two *P. aeruginosa* strains could be attributed to the differences in the Type IV pilus or the fimL genes between them [48]. However additional studies are warranted to characterize the binding profiles of these two pseudomonas strains. There are 228 genes present in USA300 (MRSA M05-86) strain but not in USA100 (MRSA M05-35) of the *Staphylococcus aureus*[49]. Lack of cell surface adhesion genes such as fnbA, fnbB and ebh in USA100 but their presence in the USA300 [49] could be the reasoning behind the binding activity differences of MRSA-35 and MRSA-86 on collagen and hyaluronan surfaces.

### Co-culture biofilms possess less biomass, denoting a possible competitive interaction between *P. aeruginosa* and MRSA

**Figure 2:**
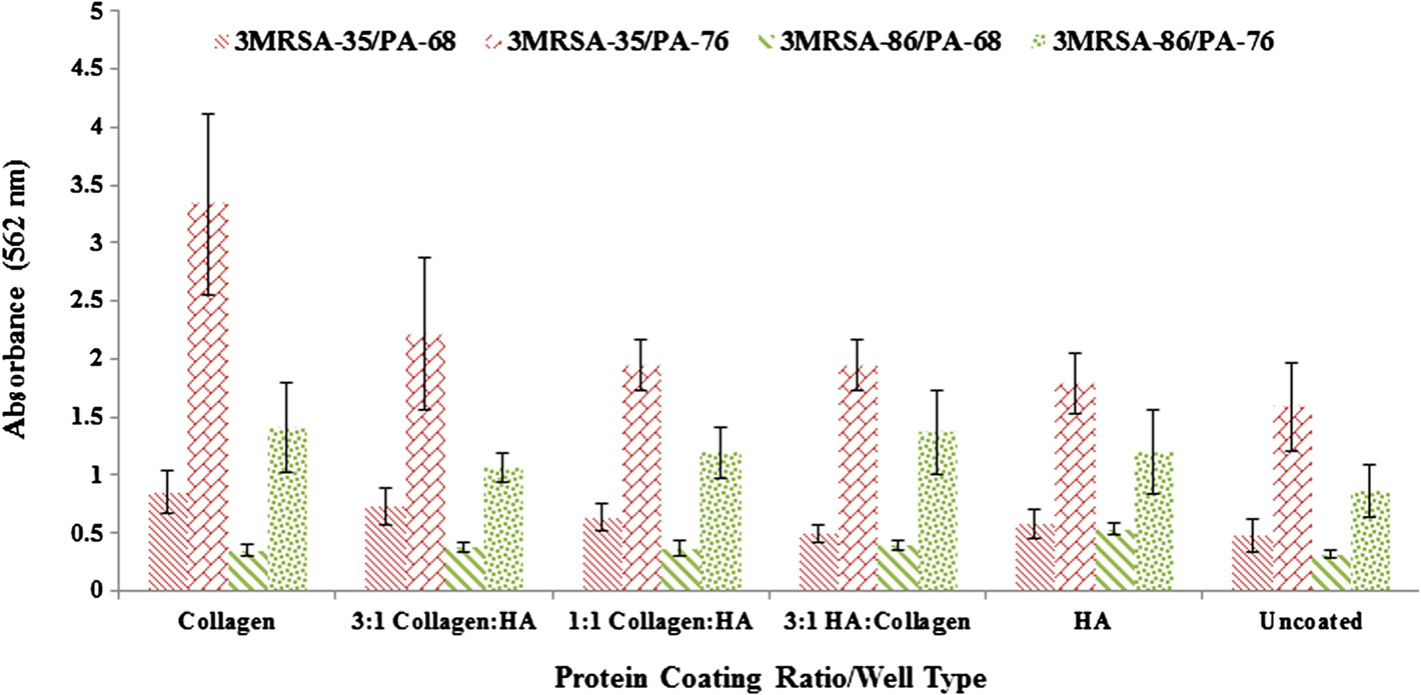
**Biofilm production in 1:1 co-cultures after 24 hours.** Biofilm production as observed at OD = 562 nm after crystal violet microtiter plate assay among *P. aeruginosa* (PA-68, PA-76) and MRSA (MRSA-35, MRSA-86). Co-cultures included PA-68/MRSA-35, PA-68/MRSA-86, PA-76/MRSA-35, and PA-76/MRSA-35. The various protein ratios/well coatings are shown in the x-axis with HA representing hyaluronan. Error bars shown represent the 1 standard deviation values for each sample.

We grew co-cultures of *P. aeruginosa* and MRSA to determine the impact on biofilm formation. 1:1 co-cultures were examined first (Figure 2). These were grown in coated and uncoated wells to assess the impact of coating on biofilm formation. Compared to *P. aeruginosa* and MRSA-35 (the highest biofilm producing microbes/strains in monoculture), all 1:1 co-cultures produced significantly less biofilm when comparing similar coating types (P < 0.05) with the exception of the MRSA-35 in the 1:1 collagen:hyaluronan compared with the PA-76/MRSA-35 co-culture in the 1:1 collagen:hyaluronan well (P = 0.53).

Between the various well coatings, no significant difference in biofilm biomass was observed between any of the wells in the PA-68/MRSA-35 and PA-68/MRSA-86 co cultures. Among the PA76/MRSA-35 co-culture, the collagen coating led to significantly higher biofilm biomass compared to the hyaluronan and uncoated wells (P = 0.04 and P = 0.001 respectively). This was also observed between the 3:1 collagen:hyaluronan and hyaluronan and uncoated wells (P = 0.04 and P = 0.002 respectively). It was also observed that the 1:1 collagen: hyaluronan well led to significantly higher biofilm biomass compared to the 3:1 collagen: hyaluronan, 3:1 hyaluronan:collagen, hyaluronan, and uncoated wells for the PA76/MRSA-35 co-culture (P < 0.05). Overall, the 1:1 collagen: hyaluronan well led to the highest biofilm biomass in the PA-76/MRSA-35 co-culture. Among the PA-76/MRSA-86 co-culture, significantly less biofilm biomass was produced in the hyaluronan well compared to the collagen well (P = 0.01).

These results imply that coating type was less relevant to biofilm production in 1:1 co-cultures as competition effects between *P. aeruginosa* and MRSA likely dominated and subsequently hindered biofilm production. It has been established that *P. aeruginosa*, *in-vitro*, negatively affects the attachment and growth of staphylococcal species, [43,50-52] something that could also be reflected in decreased biofilm production. These results are in keeping with the expected outcomes. It has been shown that in co-culture, both *S. aureus* and PA produce ‘thinner’ biofilms compared to their mono-culture biofilms [39]. This points to a competitive interaction between the two species, likely for nutrients and co-factors, such as iron, as well as for a niche to occupy, as they may compete for binding sites. The production and release of chemical signals, metabolites, and volatile compounds from *P. aeruginosa* have been implicated in affecting the growth and biofilm production of other microbes [53]. Most notably cyanide-like compounds such as pyocyanin, have been implicated in arresting *S. aureus* growth through inhibition of cellular cytochromes necessary for *S. aureus’* respiratory cycle [39,54]. These compounds can become concentrated in closed *in vitro* experiments (microtiter plate well) leading to the decreased viability of *S. aureus*[54]. Even though *P. aeruginosa* inhibits MRSA growth, it does not completely prevent it from forming biofilms as these two microbes are commonly seen together in biofilms [43]. It has been shown that *P. aeruginosa* type IV pili are essential for the process of *S. aureus* microcolony formation, an important component of biofilm adherence [43]. *P. aeruginosa* has also been shown to protect *S. aureus* against phagocytic cells such as *Dictyostelium discoideum* in co-culture biofilms [43].

**Figure 3:**
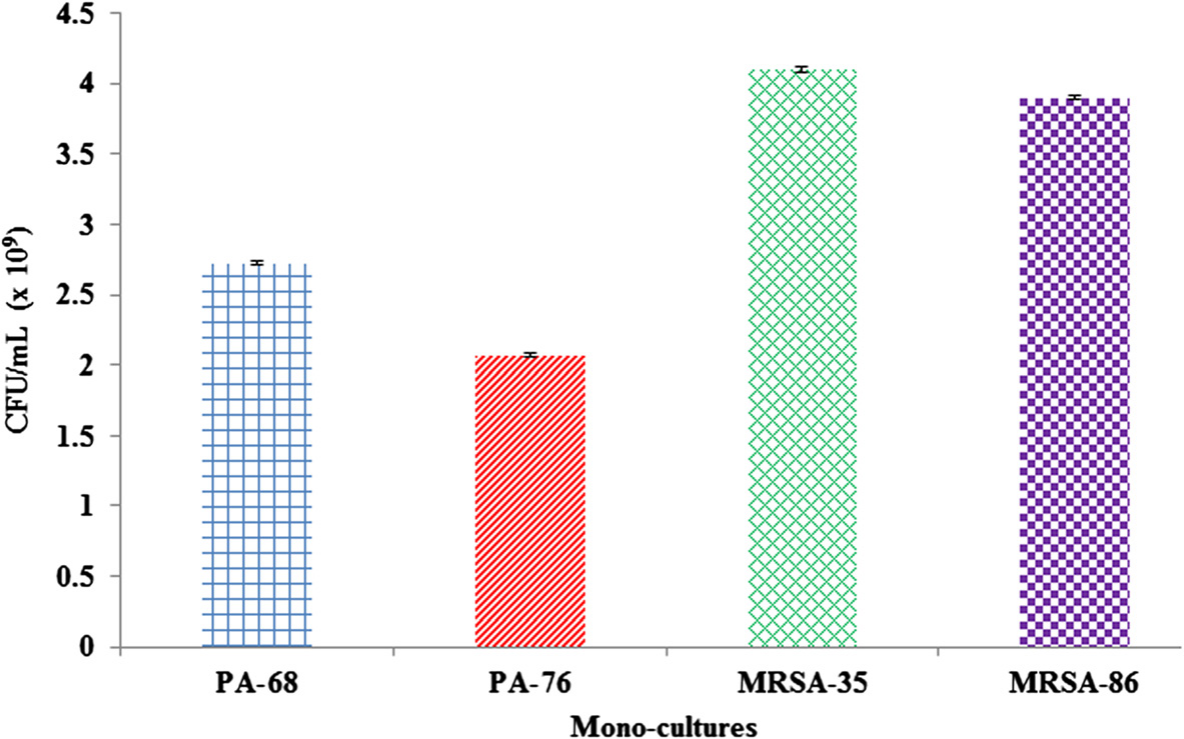
**Biofilm production in 3:1 MRSA:*P. aeruginosa *co-cultures after 24 hours.** Biofilm production as observed at OD = 562 nm after crystal violet microtiter plate assay among *P. aeruginosa* (PA-68, PA-76) and MRSA (MRSA-35, MRSA-86). 3:1 Co-cultures included MRSA-35/PA-68, MRSA-35/PA-76, MRSA-86/PA-68, and MRSA-86/PA-76. The various protein ratios/well coatings are shown in the x-axis with HA representing hyaluronan. Error bars shown represent the 1 standard deviation values for each sample.

After determining that a 1:1 ratio of *P. aeruginosa* to MRSA inhibited biofilm formation, we tried a 3:1 co-culture of the MRSA: *P. aeruginosa* to determine if a greater MRSA cell number could help to overcome these inhibitory effects (Figure 3). Biofilm formation was reduced in 3:1 cultures compared to mono-culture regardless of well coating. Compared to 1:1 co-culture biofilms, the 3:1 co-cultures showed few significant differences in the amount of biofilm biomass. Exceptions included a significant decrease in biofilm biomass between the PA-68/MRSA-35 co-culture and the 3:1 MRSA-35/PA-68 co-culture in the 3:1 hyaluronan: collagen wells (P = 0.04). Among the 3:1 MRSA-35/PA-76 co-culture a significant increase in biofilm biomass was observed compared to the PA-76/MRSA-35 co-culture in the collagen wells (P = 0.02). Other notable difference occurred between the 3:1 MRSA-86/PA-68 and PA-68/MRSA-86 co-cultures, with a significant decrease in biofilm formation among the 3:1 collagen:hyaluronan, 1:1 collagen/hyaluronan, 3:1 hyaluronan:collagen, and uncoated wells in the 3:1 co-culture (P < 0.05). Even though some significant differences existed, there was no overall trend that led us to believe there was an overall difference in biofilm biomass production between the two co-culture types.

Similar to the 1:1 co-cultures, in the 3:1 co-cultures, no unifying trend was observed in which one particular well coating led to significantly higher biomass even though some statistically significant differences existed. Again, similar to the 1:1 co-cultures, these results imply that the coating type was less relevant to biofilm production in 1:1 co-cultures as the competition effects between *P. aeruginosa* and MRSA likely dominated and subsequently hindered biofilm production.

### Co-culturing led to a decrease in the number of viable *P. aeruginosa* and MRSA cells

**Figure 4:**
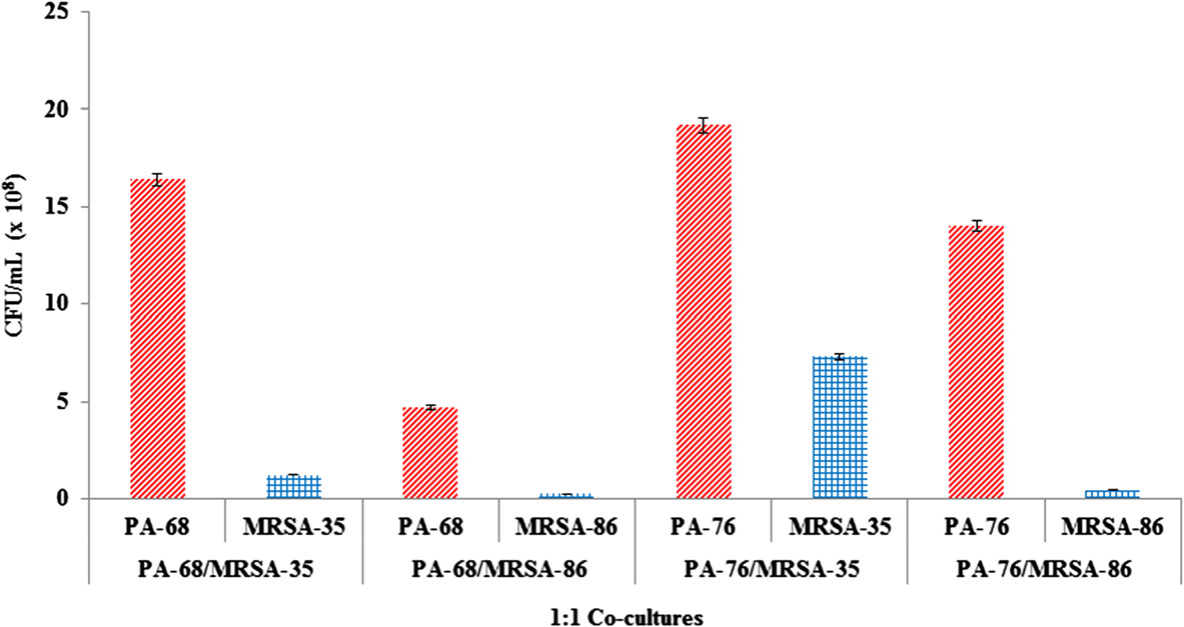
**Quantitative bacteriology of mono-culture P. *aeruginosa *and MRSA biofilm.** PA-68, PA-76, MRSA-35, and MRSA-86 CFU/mL values were quantified from growth in mono-cultures to be compared and contrasted with CFU/mL values in 1:1 and 3:1 MRSA:PA co-cultures. Both MRSA strains showed higher CFU/mL values than P. *aeruginosa* strains. Collectively the CFU/mL counts were, from greatest to least, MRSA-35 (4.1 x 109 CFU/mL), MRSA-86 (3.9 x 109 CFU/mL), PA-68 (2.73 x 109 CFU/mL), and PA-76 (2.07 x 109 CFU/mL).

**Figure 5:**
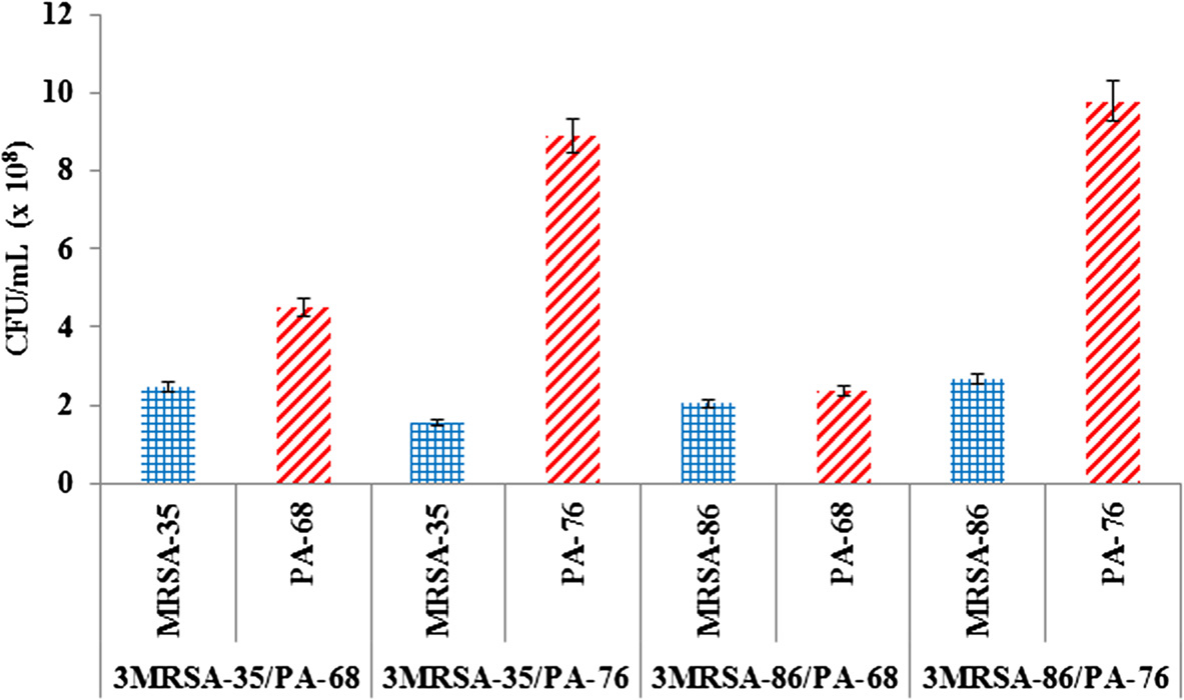
**1:1 Co-cultures show decreases in P. aeruginosa and MRSA counts with MRSA being the most affected.** Large decreases in CFU/mL counts were observed for both P. *aeruginosa* and MRSA upon co-culturing, with the largest decreases occurring for MRSA. Overall, PA-68 showed 1.66 and 5.81 magnitude decreases in CFU/mL counts upon co-culturing with MRSA-35 and MRSA-86 respectively. PA-76 showed 1.08 and 1.48 magnitude decreases in CFU/mL upon co-culturing with MRSA-35 and MRSA-86. MRSA-35 showed 33.33 and 5.61 magnitude decreases in CFU/mL counts when co-cultured with PA-68 and PA-76 respectively. MRSA-86 showed 157.89 and 88.64 magnitude decreases in CFU/mL counts when co-cultured with PA-68 and PA-76 respectively.

**Figure 6:**
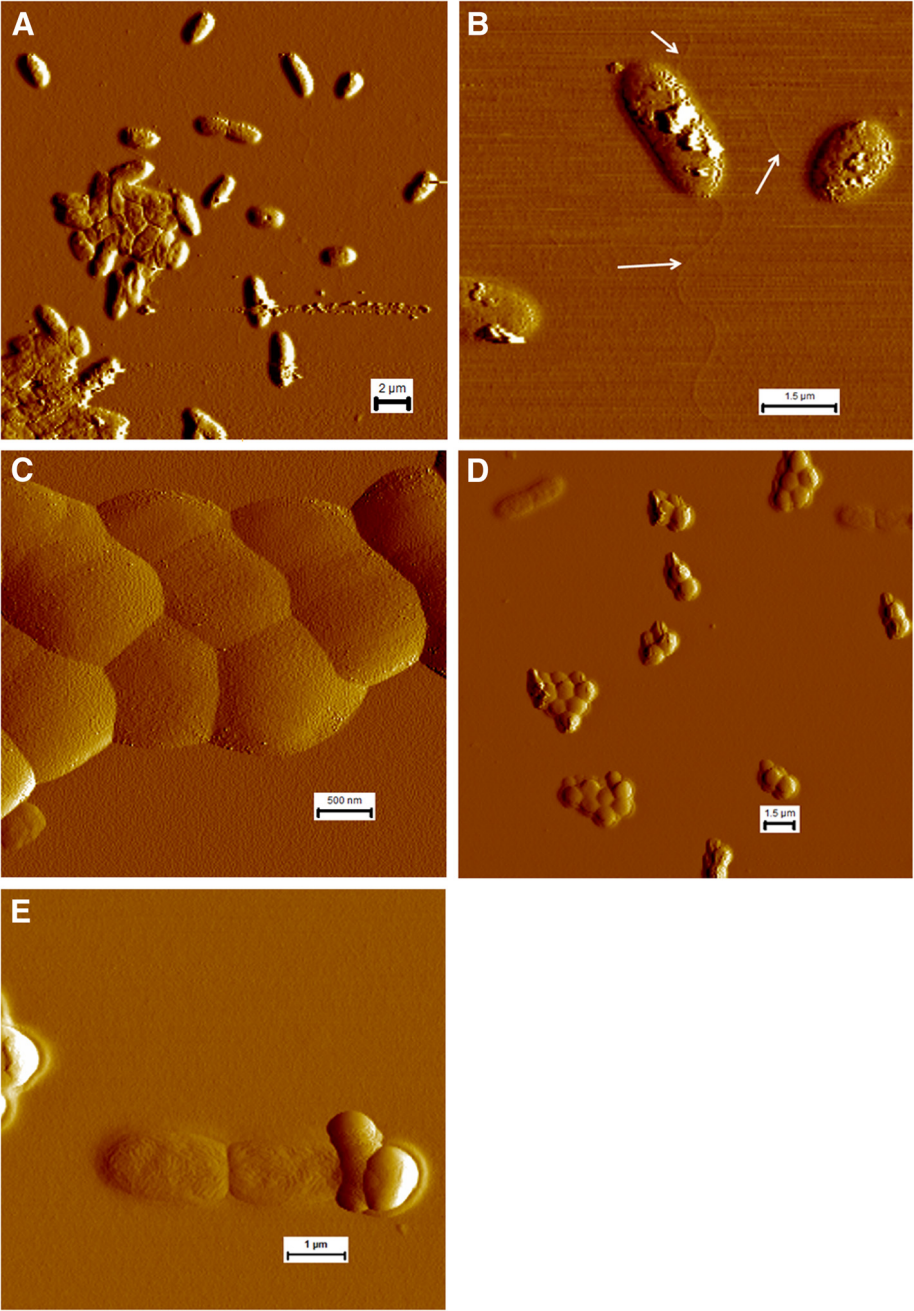
**3:1 Co-cultures show decreases in both *P. aeruginosa *and MRSA cell counts, with MRSA being less affected compared to 1:1 co-cultures.** Compared to mono-culture counts, decrease in CFU/mL were observed for both *P. aeruginosa* and MRSA upon co-culturing in 3:1 MRSA:PA ratios. MRSA counts were shown to be higher than after incubation as 3:1 co-cultures when compared to 1:1 co-culture counts. MRSA cell counts were lower than *P. aeruginosa* cell counts in all 3:1 co-culture combinations.

A selective dilution plating experiment was performed in order to determine the effects of co-culturing on cell numbers (Figures 4, 5 and 6). *P. aeruginosa* and MRSA both grew well in mono-cultures, with MRSA strains showing higher CFU/mL counts. From this it is apparent that MRSA grew to higher numbers after 24 hours than PA. Co-culturing (in 1:1 and 3:1 MRSA:PA ratios) led to large decrease in microbial CFU/mL values with MRSA appearing to be the most effected from co-culturing. Increasing the inoculum count of MRSA to 3x that of *P. aeruginosa* led to increased MRSA counts when compared to 1:1 co-cultures; however, higher MRSA inoculations did not bring MRSA counts back to mono-culture levels. Likewise, *P. aeruginosa* cell counts were shown to be more affected in 3:1 co-cultures. These results indicate that upon co-culturing microbial numbers for both species decrease significantly with MRSA being affected the most. The competitive effects between these microbes leads to a significant decreases in viable cell counts and may provide another explanation for the poor biofilm production seen in 1:1 and 3:1 MRSA:PA co-cultures (Figures 2 and 3). The reduction in MRSA cells upon co-culturing indicates that *P. aeruginosa* greatly affects MRSA cell viability. Although *P. aerugionsa* grew slowly than MRSA in mono-culture, *P. aeruginosa* cell counts were less affected from co-culturing.

### Defects in biofilm co-culture do not appear to be the result of cell lysis

**Figure 7:**
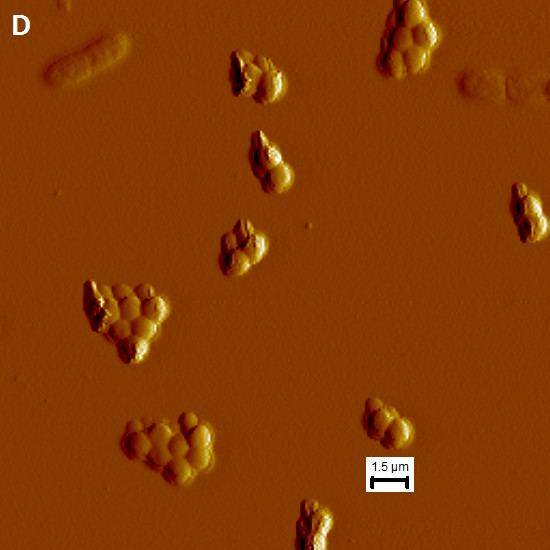
**Atomic force microscopy of *P. aeruginosa *and MRSA in mono- and co-culture on gelatin-coated mica. (A & B)** Showing overview of *P. aeruginosa* BK-76 mono-culture and two individual cells with flagella **(C)** showing MRSA M05-35 cell aggregation in mono-culture **(D & E)** showing co-culturing of *P. aeruginosa* BK-76 + MRSA M05-35. **(D)** Shows an overview of the co-culture with *P. aeruginosa* BK-76 single and attached cells. **(E)** Shows two attached *P. aerugionsa* BK-76 cells ‘attacking’ a single MRSA M05-35 cell with the appearance of spilled cytoplasmic contents.

We obtained atomic force microscopy (AFM) images of PA-76, MRSA-35, and a co-culture of PA-76/MRSA-35 biofilms, as these strains/combinations produced the greatest overall biofilm biomasses. We used imaging to monitor the overall appearance of the cells and integrity of their membranes to assess whether molecules produced in co-culture were damaging the cells and/or causing apoptosis. In the PA-76 mono-culture, the cell membranes were intact with little to no damage to the cells (Figure 7). In terms of cell morphology, there were no obvious defects in cell shape or size for mono-culture grown *P. aeruginosa* cells. *P. aeruginosa* was seen as either single cells, doubles (attached in some cases, maybe in the midst of cell division), and as densely packed aggregates of 5 or more cells. Flagella were observed upon closer examination of single cells. For MRSA-86 mono-culture, typical *Staphylococcus* clusters were identified, with the average cluster size being 2–4 cocci (Figure 7). Little to no membrane damage was observed.

For the PA-76/MRSA-35 co-culture PA-76 was found as single cells or as doubles (Figure 7). No large PA-76 cellular aggregates were noted, as for mono-cultures. The membranes of PA cells were largely intact with little to no apparent damage. Among the images collected, we were able to find a PA-76 cell that was associated with a MRSA-35 that had clearly lysed, possibly as a result of the interaction with *P. aeruginosa*. Other than this occurrence, all MRSA-35 cells appeared in aggregates of two or more cells with little to no membrane damage. No cell aggregates were observed that contained both PA-76 and MRSA-35, other than the one frame with the lysed MRSA-35 cell. Thus, it would appear that the competitive interaction that exists between these cells, which likely reduced co-culture biofilm formation, operates outside of simple biocidal mechanisms.

Study of the *P. aeruginosa* isolates from dogs has the potential to advance the understanding of both human and animal wound infection pathogenesis, and contribute to the development of animal models that may resemble spontaneous human infection. The studied *P. aeruginosa* isolates from dogs have similar pathogenic phenotypes and genotypes of human relevant strains. Hence, it is reasonable to extrapolate the results obtained to humans.

## Conclusions

The microtiter plate assay provides a good initial model for testing the effects of protein coating on the production of biofilm. In this study, it was observed that protein coating led to enhanced biofilm production in monoculture biofilms. This trend was not observed in co-culture. Among the varying ratios of collagen: HA coatings, few definable trends were found in the mono- and co-cultures. No single combination resulted in a dramatic increase or decrease in biofilm formation, although collagen alone did seem to produce slightly more biofilm biomass on average. Co-culturing led to a decrease in biofilm production compared to either mono-culture biofilm. Among the co-cultures, no observable difference in biofilm was produced in the 3:1 MRSA: PA co-culture compared to the 1:1 co-cultures, aside from a few exceptions. The Atomic Force Microscopy imaging was able to reveal changes in cell packing between the mono- and co-culturing of PA-76 and MRSA-35 and showed that the competitive interaction between these *P. aeruginosa* and MRSA likely operates outside of a simple biocidal mechanism.

## Competing interests

There were no financial competing interests or conflicts of interest throughout all research or submission of this manuscript.

## Authors’ contributions

EB designed portions of the study, conducted all experiments, and wrote the manuscript. SN coordinated the project, designed portions of the study, and helped revise and draft the manuscript. SW interpreted the data and critically revised the manuscript. All authors have read and approved the final manuscript.
